# Colorado's Ryan White screening, brief intervention, and referral to treatment collaborative project to address substance use in HIV/AIDS case management and health-care settings

**DOI:** 10.1186/1940-0640-7-S1-A73

**Published:** 2012-10-09

**Authors:** Leigh Fischer

**Affiliations:** 1 Peer Assistance Services, Screening, Brief Intervention, and Referral to Treatment (SBIRT) Colorado, Denver, CO, USA

## 

This presentation discusses screening, brief intervention, and referral to treatment (SBIRT) for substance use within HIV/AIDS treatment and care programs and lessons learned from Colorado's Ryan White SBIRT collaborative project. Evidence demonstrates that SBIRT in primary care settings is effective in changing behavior and preventing adverse outcomes attributable to alcohol and other drugs. Studies also show people living with HIV are more likely to experience substance abuse problems than the general population, and early detection offsets the negative ramifications, including poor treatment adherence. Despite the linkage between substance use and HIV, screening and brief intervention protocols have not been readily adopted in HIV/AIDS services in the United States. In order to introduce SBIRT procedures tailored for HIV/AIDS care, Colorado implemented a collaboration between the state’s SBIRT initiative and its Ryan White Part B HIV treatment and care program. Of 2500 patients screened, 31% received a brief intervention for risky alcohol, tobacco, or drug use, and 23% were referred for therapy or specialized treatment. Program evaluation findings gathered from focus groups and patient and provider surveys indicate that SBIRT can be successfully integrated into HIV treatment and care to address risky substance use. We explore the barriers to implementing SBIRT in HIV care and identify the administrative and policy considerations necessary for effective implementation. Recommendations are made for standardizing SBIRT in HIV care (applying a systematic approach to screening; training providers to conduct brief interventions; establishing a referral network; and integrating SBIRT with adherence and retention efforts).

